
JOURNAL INFORMATION
==============================
NLM Title Abbreviation: Atlanta Med Surg J (1884)
NLM Journal ID: 101251623

Atlanta Medical and Surgical Journal (1884)

Publisher: Biomedical Journal Digitization Project

ARTICLE INFORMATION
==============================
PMCID: PMC9201848
Article version: 1
Subjects: Cover
Print publication date: 1899 Jan
Volume: 15
Volume Series: New Ser
Issue: 11
License: This work is free of known copyright restrictions. For more information, please see PMC Back Issue Digitization.
License URL: https://creativecommons.org/publicdomain/mark/1.0/

==============================

PHILLIPS' EMULSION exhibits the highest degree of excellence in emulsionizing Cod Liver Oil. 50 per cent. finest Norway Oil—in minute subdivision—emulsified by Pancreatine—combined with the Wheat Phosphates (PHILLIPS'). Acid reaction, precluding saponification.

PALATABLE—PERMANENT.

Miscible in Water, Milk, Wine, etc. Prescribe PHILLIPS'.

THE CHAS. H. PHILLIPS CHEMICAL CO., 77 Pine St., New York.

Please mention this Journal.

LIBRARY Indexed S. G. O.

ESTABLISHED 1855. SUBSCRIPTION $1.00.

LIBRARY SURGEON GENERAL'S OFFICE JAN.-12-1899

### ATLANTA MEDICAL AND SURGICAL JOURNAL.

VOLUME XV NEW SERIES. ATLANTA, GA., JANUARY, 1899. No. 11.

NEURASTHENIA, NERVE STARVATION AND BLOOD POVERTY

are the conditions in which Mercauro has produced desired results, never before secured by me in a practice covering over twenty years.

A. M. Owen, M. D.

Ex Member Judicial Council Amer.Med.Assn.
Ex Pres. Miss. Valley Med. Assn.
[illegible]
